# Improved image quality and T-staging accuracy using FOCUS-MUSE DWI in gastric cancer: a prospective comparison with SS-EPI

**DOI:** 10.1186/s41747-026-00755-6

**Published:** 2026-06-17

**Authors:** Qiong Li, Ya-Jun Hou, Qian-Yun Jiang, Jie Xu, Qiu-Xia Feng, Lei Yuan, Pu-Yeh Wu, Chen-Jiang Wu, Xi-Sheng Liu, Na-Na Sun

**Affiliations:** 1 Department of Radiology, The First Affiliated Hospital of Nanjing Medical University, Nanjing, China; 2 MR Research Collaboration Team, GE Healthcare, Beijing, China

**Keywords:** Diagnostic imaging, Diffusion magnetic resonance imaging, Echo-planar imaging, Neoplasm staging, Stomach neoplasms

## Abstract

**Objective:**

To compare the image quality and diagnostic value of field-of-view optimized and constrained undistorted single-shot multiplexed sensitivity-encoding diffusion-weighted imaging (FOCUS-MUSE DWI) with single-shot echo-planar imaging (SS-EPI) DWI in gastric cancer.

**Materials and methods:**

Patients with biopsy-proven gastric cancer who underwent FOCUS-MUSE and SS-EPI DWI were enrolled in this prospective study between February and October 2024. Qualitative image quality was evaluated by two radiologists using a standardized five-point scale. Quantitative comparisons of signal-to-noise ratio (SNR), contrast-to-noise ratio (CNR), and apparent diffusion coefficient (ADC) were conducted for all measurable lesions. Preoperative T-staging accuracy was compared between the two techniques, with pathological or surgical findings as the reference. Agreement between estimations was evaluated using weighted kappa values and Bland–Altman plots. Pairwise differences between sequences were assessed using the Wilcoxon signed-rank and McNemar tests.

**Results:**

Among 177 patients (age 65.4 ± 9.8 years, mean ± standard deviation; 133 males), FOCUS-MUSE DWI outperformed SS-EPI DWI in lesion conspicuity and overall image quality (median scores, 5 *versus* 4; both *p* < 0.001). For 148 patients with measurable lesions, FOCUS-MUSE yielded higher SNR (33.6 and 34.6 *versus* 26.9 and 26.9) and CNR (7.1 and 7.7 *versus* 5.3 and 5.9; all *p* < 0.001). Among 128 patients with pathologically T1–4a (pT1–T4a), surgically T4b (sT4b), or pathologically T4b (pT4b), T-staging accuracy was higher with FOCUS-MUSE (64.8% and 63.3%) than SS-EPI (44.5% and 47.7%; both *p* < 0.001). Intra- and inter-reader agreement was good to excellent.

**Conclusions:**

FOCUS-MUSE DWI demonstrated higher image quality and T-staging accuracy than SS-EPI DWI in gastric cancer.

**Relevance statement:**

FOCUS-MUSE DWI provides improved image quality compared with conventional SS-EPI DWI and enables more accurate preoperative T staging in gastric cancer. These findings support the potential role of advanced DWI techniques in improving local tumor assessment.

**Key Points:**

- FOCUS-MUSE DWI demonstrated higher image quality compared to SS-EPI DWI in gastric cancer.
- FOCUS-MUSE DWI significantly improved preoperative T-staging accuracy over SS-EPI DWI.
- This advanced DWI technique shows potential to enhance preoperative risk stratification and surgical planning.

**Graphical Abstract:**

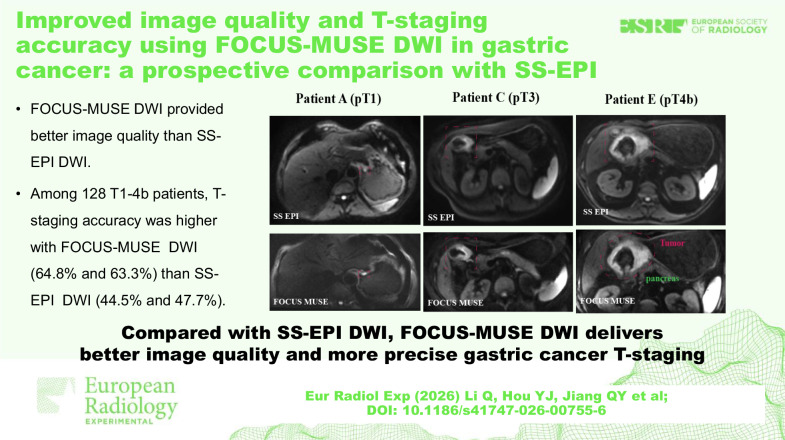

## Background

Although the incidence of gastric cancer has declined, it remains a leading cause of cancer-related mortality worldwide, particularly in high-prevalence regions such as East Asia [1]. Accurate assessment of tumor invasion depth (T stage) is critical for guiding management. This distinction, for example, between tumors amenable to resection (T2–4a) and those involving adjacent structures (T4b), directly influences the choice between curative surgery, neoadjuvant therapy, or multi-organ resection, thereby substantially affecting patient outcomes [2].

CT remains the first-line modality for initial staging owing to its widespread availability and efficiency in detecting distant metastases. However, its ability for precise local-regional T stage is limited by suboptimal soft-tissue contrast, which frequently impedes reliable differentiation of individual gastric wall layers. Consequently, multiparametric magnetic resonance imaging (MRI) is increasingly evaluated as a complementary option for preoperative staging. Comparative studies indicate that MRI, particularly when incorporating diffusion-weighted imaging (DWI) and dynamic contrast-enhanced sequences, outperforms CT in T-stage evaluation, whereas T2-weighted imaging shows more variable and often inferior diagnostic performance [3, 4]. Within this optimized framework, DWI has become a cornerstone functional sequence [5, 6]. By quantifying the mobility of water molecules, DWI provides unique insights into tissue microstructure, and the derived apparent diffusion coefficient (ADC) has shown potential as a biomarker for tumor aggressiveness, treatment response, and prognosis [7–10].

The conventional DWI technique, single-shot echo-planar imaging (SS-EPI), is widely employed but exhibits significant limitations in the upper abdomen, including low spatial resolution and pronounced susceptibility artifacts from air-tissue interfaces. These artifacts degrade image quality and compromise staging confidence [11]. Such drawbacks have prompted the development of advanced imaging techniques to enhance spatial resolution and mitigate artifacts.

Reduced field-of-view DWI techniques, such as field-of-view optimized and constrained undistorted single-shot (FOCUS) DWI, improve image quality by selectively exciting a smaller targeted region, thereby shortening the readout and minimizing distortions [12]. When combined with multiplexed sensitivity-encoding (MUSE), FOCUS-MUSE DWI employs a multi-shot acquisition strategy in the phase-encoding directions, yielding images with high spatial resolution, excellent spatial fidelity, and reduced motion-induced phase errors that plague SS-EPI [13, 14]. Previous studies have confirmed that FOCUS-MUSE DWI outperforms conventional methods in evaluating diseases in anatomically complex regions like the lung, pancreas, and orbit, with improved lesion conspicuity and diagnostic confidence [15–17].

Despite these technical advances, the application of FOCUS-MUSE DWI in gastric cancer remains largely underexplored. We hypothesize that the implementation of FOCUS-MUSE DWI can improve image quality and staging accuracy relative to SS-EPI DWI. This prospective study aims to systematically compare FOCUS-MUSE and SS-EPI DWI in patients with gastric cancer with respect to image quality, ADC measurements, and T-staging performance.

## Methods

### Study subjects and pipeline

This exploratory, prospective, single-center study consecutively recruited patients with biopsy-confirmed gastric cancer who were referred for preoperative MRI between February and October 2024. The inclusion criteria were: (1) histopathologically confirmed gastric cancer; and (2) completion of a standardized MRI protocol. Exclusion criteria were: (1) inability to provide informed consent; (2) general contraindications to MRI; and/or (3) examination on a scanner lacking the FOCUS-MUSE sequence. As an exploratory study without prior data for a formal power calculation, the sample size was determined by consecutively enrolling all eligible patients during the predefined study period. This study was approved by the Ethics Committee of our hospital (Approval No. 2021-SR-475).

**Fig. 1:**
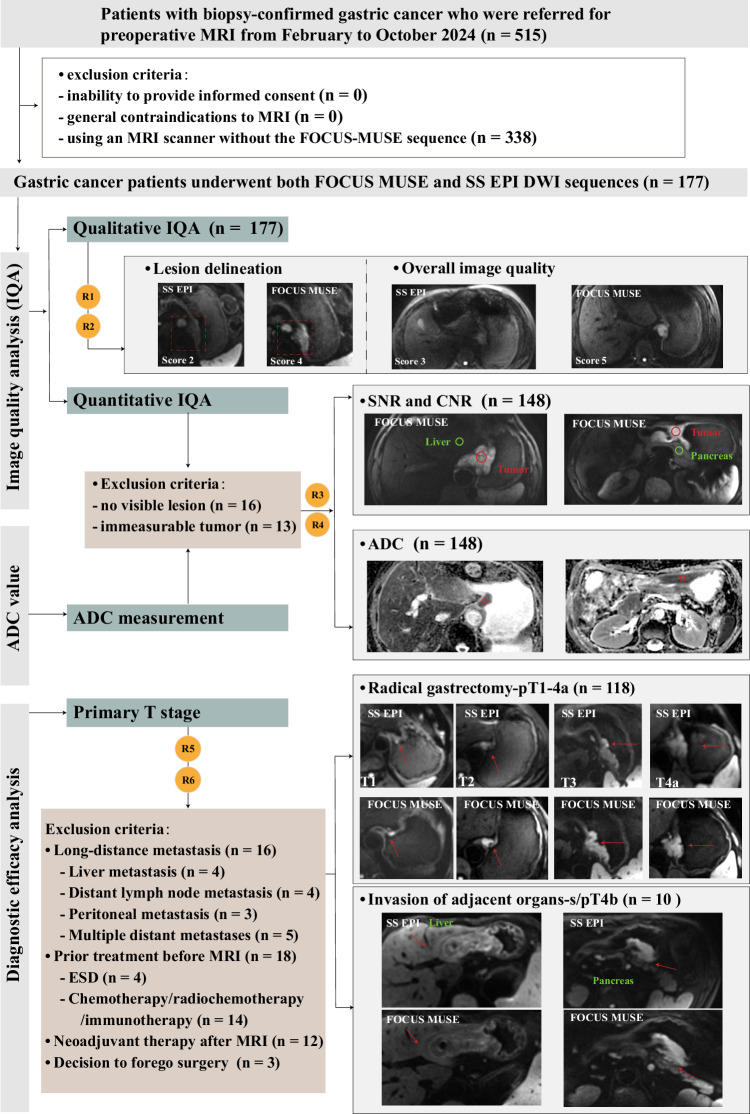
Flowchart of the study. ADC, Apparent diffusion coefficient; CNR, Contrast-to-noise ratio; DWI, Diffusion-weighted imaging; ESD, Endoscopic submucosal dissection; FOCUS-MUSE, Field-of-view optimized and constrained undistorted single-shot multiplexed sensitivity-encoding; IQS, Image quality score; SNR, Signal-to-noise ratio; SS-EPI, Single-shot echo-planar imaging

The study consisted of three sequential, blinded analyses (Fig. 1):

1. Qualitative image analysis—All patients who underwent both FOCUS-MUSE and SS-EPI DWI were included; image quality scores for lesion delineation and overall image quality were assessed;
2. Quantitative image analysis—Patients were excluded if the lesion was not visible or not measurable for region-of-interest (ROI) placement on either DWI sequence; for the remaining patients, signal-to-noise ratio (SNR), contrast-to-noise ratio (CNR), and ADC values were calculated;
3. Diagnostic efficacy assessment—The cohort for T-staging accuracy assessment was derived from the patients who had undergone qualitative image analysis. From this group, we only included those who subsequently underwent radical gastrectomy or laparoscopic exploration within 4 weeks of the MRI examination. Patients were excluded if they had distant metastasis, received any anti-tumor treatment before MRI, initiated neoadjuvant therapy after MRI, or declined surgery. This cohort included all surgically confirmed tumors, thereby assessing diagnostic performance even for lesions invisible/unmeasurable on DWI.

Six abdominal radiologists with varying levels of experience (Supplementary Table S1) participated in the study. All had undergone formal training in gastrointestinal MRI. To mitigate recall and anchoring biases across different assessment tasks, readers were assigned fixed roles: Readers 1 and 2 performed qualitative assessments; Readers 3 and 4 conducted quantitative measurements; and Readers 5 and 6 determined the T-stage. Prior to the study reads, all readers completed a standardized calibration session. Readers were informed that each case had biopsy-confirmed gastric cancer and were provided with the tumor location from clinical or endoscopic documentation. No other clinical information was available. They were blinded to all other clinical data, other MRI sequences, CT images, and the final pathological or surgical findings.

### Image acquisition

MRI examinations were performed using a 3-T scanner (SIGNA™ Premier; GE Healthcare). Before the scan, patients were instructed to drink 800–1,000 mL of water to distend the stomach. If suitable, they received 20 mg of anisodamine intramuscularly to minimize gastrointestinal motion. In case of anisodamine contraindications, 40 mg of drotaverine hydrochloride was administered. Depending on tumor location, patients were scanned in either the supine or prone position to minimize air-related artifacts. The imaging protocol included routine structural imaging, SS-EPI DWI, and FOCUS-MUSE DWI. Detailed acquisition parameters are provided in Supplementary Table S2.

### Qualitative image analysis

Two radiologists (Readers 1 and 2, with 3 and 5 years of clinical experience, respectively) independently scored the image quality for both DWI (*b* = 800 s/mm^2^) methods using a 5-point Likert scale. Lesion detectability and overall image quality were evaluated, ranging from 1 (non-diagnostic/poor) to 5 (high confidence/excellent). If the lesion was not visualized on a given implementation, conspicuity for that implementation was recorded as non-diagnostic per the study’s ordinal scale, whereas the alternate implementation was scored per protocol. To avoid recall bias, a 2-week washout period was enforced, and the second assessment was randomized.

### Quantitative image analysis

SNR and CNR were independently measured and calculated by two radiologists (Readers 3 and 4, with 4 and 5 years of clinical experience, respectively) who were blinded to image and patient information. Specifically, ROIs were manually delineated on SS-EPI DWI and FOCUS-MUSE DWI images (*b* = 800 s/mm^2^), excluding necrotic regions and focusing on lesions > 5 mm in diameter (Fig. 1). Lesions that could not accommodate an ROI were marked as unmeasurable. For each measurable lesion, ROIs were manually delineated using a freehand approach on the slice showing the largest tumor area, while avoiding necrotic and visible artifacts. For each patient, ROIs were placed at matched locations on FOCUS-MUSE DWI and SS-EPI DWI to ensure consistent within-patient comparison of quantitative image analysis. The mean and standard deviation (SD) of signal intensity (SI) within the tumor ROI were recorded as SI_tumor_ and SD_tumor_, respectively. ROIs in adjacent organs (*e.g*., liver or pancreas) were also defined on the same section to obtain the mean (SI_liver_ or SI_pancreas_) and standard deviation (SD_liver_ or SI_pancreas_) of SI. SNR and CNR were calculated using the following formulas:

$${{\rm{SNR}}}={{{\rm{SI}}}}_{{{\rm{tumor}}}}/{{{\rm{SD}}}}_{{{\rm{liver}}}\; {{\rm{or}}}\; {{\rm{pancreas}}}}$$

$${{\rm{CNR}}}=({{{\rm{SI}}}}_{{{\rm{tumor}}}}-{{{\rm{SI}}}}_{{{\rm{liver}}}\; {{\rm{or}}}\; {{\rm{pancreas}}}})/\sqrt{{({{SD}}_{{{\rm{tumor}}}})}^{2}+{({{{\rm{SD}}}}_{{{\rm{Liver}}}\; {{\rm{or}}}\; {{\rm{pancreas}}}})}^{2}}$$

The apparent diffusion coefficient (ADC) was computed using a mono-exponential model based on DWI images with *b*-values of 50 and 800 s/mm^2^. The previously defined tumor ROIs were applied to ADC maps to extract mean ADC values, and re-evaluations were conducted after a 2-week washout.

### T-staging evaluation

DWI images (*b* = 800 s/mm^2^) were used for radiological T-staging (rT0, rT1, rT2, rT3, rT4a, and rT4b). rT0 tumors were defined as non-visible. The golden standards were pathologically confirmed T1–T4a (pT1–T4a), surgically confirmed T4b (sT4b), or pathologically confirmed T4b (pT4b). The sT4b staging was characterized by adjacent organ invasion on surgical findings without pathological confirmation, and pT4b staging was defined by the presence of pathologically confirmed adjacent organ invasion. Two radiologists (Readers 5 and 6, with 10 and 15 years of clinical experience, respectively) independently recorded and re-evaluated T-staging after a 2-week washout. The pathologists were blinded to the MRI results.

### Statistical analysis

Statistical analyses were performed using R (version 4.4.2) and MedCalc (version 20.2). The following comparisons and assessments were pre-specified. Categorical data were presented as numbers and percentages, while continuous data were presented as median and interquartile range. Normality was checked using the Kolmogorov-Smirnov test. Inter- and intra-rater reliability for the quantitative data, and the ADC agreement between FOCUS-MUSE and SS-EPI DWI sequences were evaluated using Bland–Altman plots. T-staging agreement was evaluated using the Weighted κ test, and the κ values were classified as follows: < 0.40, poor; 0.41–0.60, moderate; 0.61–0.80, good; ≥ 0.81, excellent. The Wilcoxon signed-rank test was used to compare the image quality scores, SNR, CNR, and ADC between FOCUS-MUSE and SS-EPI DWI, while the McNemar test assessed T-staging differences between the two sequences. *p* < 0.050 was considered statistically significant. Effect sizes were calculated using Cohen’s *d* to complement statistical significance testing and to facilitate interpretation of the magnitude of observed differences between sequences.

## Results

### Basal characteristics

From February to October 2024, the study initially recruited 515 histologically confirmed gastric cancer patients who underwent gastric MRI examination; 338 patients underwent examination on an MRI scanner without the FOCUS-MUSE sequence. No patient was excluded due to lack of informed consent or MRI contraindications. Ultimately, 177 participants (aged 65.4 ± 9.8 years, mean ± SD; 133 males) were evaluated in this study. 29 patients had unmeasurable lesions. Thus, 148 patients (aged 65.4 ± 10.2 years; 111 males) were included for quantitative analysis. 128 patients underwent radical gastrectomy or laparoscopic exploration. Among them, 118 patients were diagnosed with pT1–T4a, 4 with pT4b, and 6 with sT4b, respectively. Among the pT4b cases, two tumors invaded the pancreas, one invaded the liver, and another invaded the transverse colon. Among the sT4b cases, four invaded the pancreas, one invaded both the liver and pancreas, and one invaded the liver. The median interval between MRI examination and surgery was 3 days (range 1–20 days). The basal characteristics of the enrolled participants are summarized in Table 1.

**Table 1:** Demographic, clinical, and pathological characteristics of patients with gastric cancer in this study

Characteristic	Number (%)
Age (years)	65.4 ± 9.8*
Sex (*n* = 177)	
Male	133/177 (75.1)
Female	44/177 (24.9)
Lesion location (*n* = 177)	
Cardia or fundus	85/177 (48.0)
Body	50/177 (28.2)
Antrum or pylorus	38/177 (21.5)
Diffuse	4/177 (2.3)
Type of surgery (*n* = 128)	
Standard gastrectomy	118/128 (92.2)
Gastrectomy with combined resection of adjacent involved organs	4/128 (3.1)
Non-resectional surgery	6/128 (4.7)
Final T stage (*n* = 128)	
pT1	24/128 (18.8)
pT2	12/128 (9.4)
pT3	57/128 (44.5)
pT4a	25/128 (19.5)
pT4b	4/128 (3.1)
sT4b	6/128 (4.7)

Unless otherwise indicated, values are the number of patients, with percentage in parentheses

* Mean ± standard deviation. A total of 128 patients underwent radical gastrectomy or laparoscopic exploration

### Qualitative image analysis

**Fig. 2:**
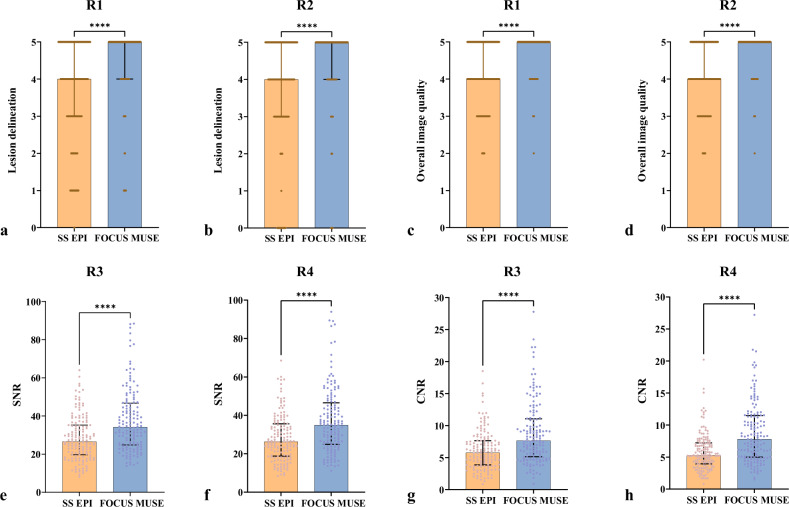
Comparison of lesion delineation, overall image quality, SNR, and CNR between SS-EPI DWI and FOCUS MUSE DWI. **a**–**d** FOCUS MUSE DWI consistently outperformed SS-EPI DWI, showing significantly higher scores for both lesion delineation (**a**, **b**) and overall image quality (**c**, **d**) across Reader 1 and Reader 2 (all *p* < 0.0001). **e**–**h** FOCUS MUSE DWI demonstrated significantly higher SNR (**e**, **f**) and CNR (**g**, **h**) compared to SS-EPI DWI (*p* < 0.0001). **** *p* < 0.0001. CNR, Contrast-to-noise ratio; DWI, Diffusion-weighted imaging; FOCUS MUSE, Field-of-view optimized and constrained undistorted single-shot multiplexed sensitivity-encoding; R, Reader; SNR, signal-to-noise ratio; SS-EPI, Single-shot echo-planar imaging

**Fig. 3:**
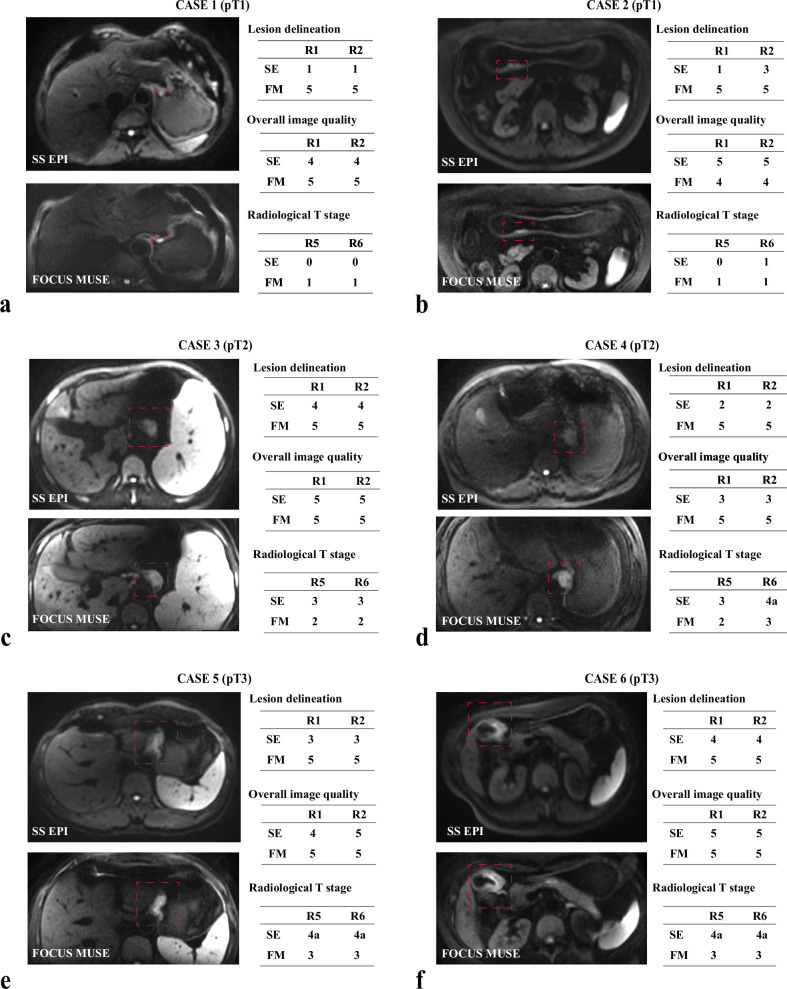
Representative cases of pathologically confirmed gastric cancer (pT1–pT3), with evaluations of lesion delineation, overall image quality, and T staging by two groups of radiologists (R1, R2 for image quality; R5, R6 for T staging). **a**, **b** pT1: In both cases, FOCUS MUSE DWI demonstrated improved lesion conspicuity compared to SS-EPI DWI. Case 1 (**a**, supine position) and Case 2 (**b**, prone position) were accurately staged as pT1 using FOCUS MUSE DWI. **c**, **d** pT2: FOCUS MUSE DWI provided clearer lesion delineation and gastric wall layers compared to SS-EPI DWI in Case 3 and Case 4 (supine position). Over-staging was observed with SS-EPI DWI for both cases. **e**, **f** pT3: In Case 5 (**e**, supine position), the tumor margin appeared unclear on SS-EPI DWI, leading to over-staging by two readers. FOCUS MUSE resolved this issue, enabling accurate staging consistent with pathological findings. For Case 6 (**f**, prone position), irregular tumor margins on SS-EPI DWI were interpreted by both readers as serosal invasion. However, the enhanced image quality of FOCUS MUSE revealed that the high and linear signal surrounding the tumor likely represented a vascular shadow, with the tumor’s serosal layer remaining intact. DWI, Diffusion-weighted imaging; FOCUS MUSE, Field-of-view optimized and constrained undistorted single-shot multiplexed sensitivity-encoding; R, Reader; SS-EPI, Single-shot echo-planar imaging

Excellent intra-reader agreement was observed for lesion delineation (κ > 0.8), while good agreement was achieved for overall image quality (κ = 0.612–0.798) (Table 2). Inter-reader agreement was good for qualitative analysis, with kappa values ranging from 0.647 to 0.754. Both Reader 1 and Reader 2 consistently rated FOCUS-MUSE DWI significantly higher than SS-EPI DWI in image quality. FOCUS-MUSE DWI exhibited significantly improved lesion delineation (median score 5 *versus* 4; *p* < 0.001) and higher overall image quality (median score 5 *versus* 4; *p* < 0.001) (Fig. 2a–d). Results of subjective image quality assessments from representative proximal (supine position) and distal (prone position) gastric scans are shown in Fig. 3. Enhanced lesion visualization, particularly in tumor conspicuity and boundary delineation, was observed with FOCUS-MUSE DWI compared to SS-EPI DWI.

**Table 2:** Intra-reader and inter-reader agreement for image quality scores

Image quality scores	Reader 1 (1st)	Reader 1 (2nd)	Reader 2 (1st)	Reader 2 (2nd)	Intra-rater (R1)^a^	Intra-rater (R2)^a^	Inter-rater^a^
Lesion delineation							
SS-EPI DWI	4 (3, 5)	4 (3, 5)	4 (3, 5)	4 (3, 5)	0.824	0.803	0.667
FOCUS-MUSE DWI	5 (3, 5)	5 (3, 5)	5 (3, 5)	5 (3, 5)	0.880	0.819	0.754
* p*-value	< 0.001	< 0.001	< 0.001	< 0.001	-	-	-
Overall image quality							
SS-EPI DWI	4 (4, 5)	4 (4, 5)	4 (4, 5)	4 (4, 5)	0.769	0.612	0.647
FOCUS-MUSE DWI	5 (5, 5)	5 (5, 5)	5 (4, 5)	5 (5, 5)	0.798	0.699	0.660
* p*-value	< 0.001	< 0.001	< 0.001	< 0.001	-	-	-

Data presented as median (interquartile range) unless specified

*DWI* Diffusion-weighted imaging, *FOCUS-MUSE* Field-of-view optimized and constrained undistorted single-shot multiplexed sensitivity-encoding, *SS-EPI* Single-shot echo-planar imaging

^a^ Weighted κ test

### Quantitative image analysis

Bland–Altman plots demonstrated excellent intra-reader agreement for SNR measurements, with minimal biases of 0.1, 0.3, 0.0, and -0.1 for the first and second readings of Readers 3 and 4, respectively (Supplementary Fig. S1a–d). The intra-reader agreement for CNR measurements was also excellent, with biases of 0.0, 0.0, -0.1, and 0.1 for the first and second readings of both readers (Supplementary Fig. S1e–h). Supplementary Fig. S2a–d further highlights the strong inter-reader agreement for SNR and CNR measurements. The SNR and CNR of FOCUS-MUSE DWI were significantly higher than those of SS-EPI DWI (*p* < 0.001, Cohen’s *d* = 0.424–0.634), as shown in Fig. 2e–h. For Reader 3, FOCUS-MUSE DWI had an SNR of 33.6 (24.8–45.2), compared with 26.9 (19.1–36.8) for SS-EPI DWI. Reader 4 reported an SNR of 34.6 (25.4–47.8) *versus* 26.9 (19.9–37.7). Similarly, FOCUS-MUSE demonstrated higher CNR than SS-EPI, with values of 7.1 (4.9–10.9) *versus* 5.3 (3.9–7.2) for Reader 3 and 7.7 (5.0–10.2) *versus* 5.9 (3.9–7.9) for Reader 4.

### ADC measurements

ADC values derived from FOCUS-MUSE and SS-EPI DWI exhibited excellent intra-reader and inter-reader agreement for both Reader 3 and Reader 4 (Supplementary Figs. S1i–l and S2e, f). Bland–Altman analysis showed minimal intra-reader bias for SS-EPI DWI (0.02 and 0.01 for Readers 3 and 4, respectively) and for FOCUS-MUSE DWI (0.0 and 0.01). Inter-reader agreement was similarly high, with negligible bias observed for both SS-EPI DWI and FOCUS-MUSE DWI (0.0 and 0.02). In addition, Bland–Altman analysis comparing ADC values between FOCUS-MUSE and SS-EPI DWI demonstrated minimal inter-sequence bias (0.05 and 0.03 for Readers 3 and 4, respectively; Supplementary Fig. S3a, b). For Reader 3, ADC values from FOCUS-MUSE DWI were slightly but significantly higher than those from SS-EPI DWI (1.35 [1.14–1.54] × 10^-3^ mm^2^/s *versus* 1.31 [1.11–1.51] × 10^-3^ mm^2^/s; *p* < 0.001; Cohen *d* = 0.383). Similarly, for Reader 4, ADC values from FOCUS-MUSE DWI were also significantly higher than SS-EPI DWI (1.34 [1.12–1.52] × 10^-3^ mm^2^/s *versus* 1.31 [1.11–1.48] × 10^-3^ mm^2^/s; *p* = 0.001; Cohen *d* = 0.288) (Supplementary Fig. S3c, d).

### Diagnostic performances of T staging

**Fig. 4:**
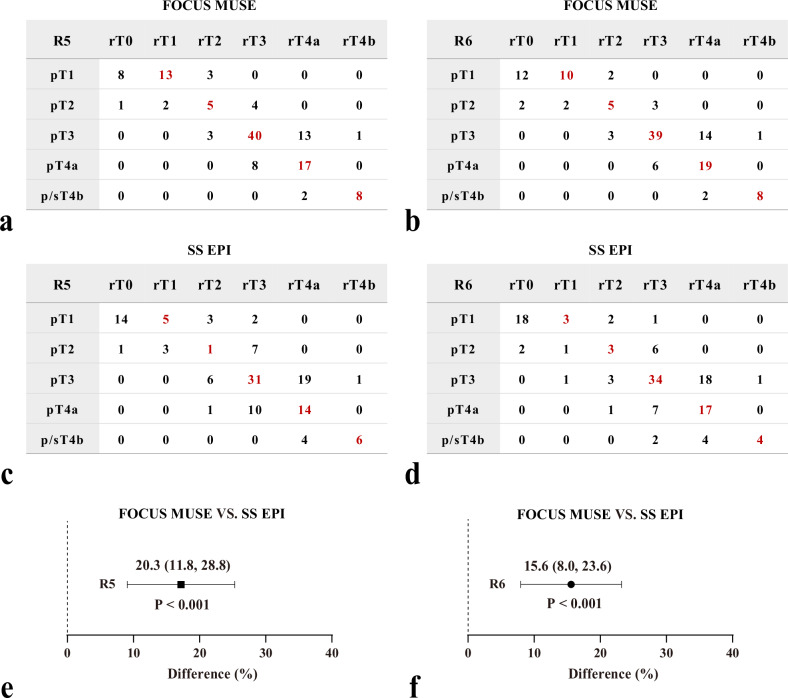
T-staging matrices and McNemar test comparing FOCUS MUSE and SS-EPI DWI. **a**–**d**: Stage classifications (pT1–p/sT4b) are plotted against radiological stages (cT0–cT4b) for FOCUS MUSE DWI (**a** R5, **b** R6) and SS-EPI DWI (**c** R5, **d** R6), with numbers in red indicating correctly staged cases. **e**, **f** Differences in the proportions of accurate staging between FOCUS MUSE and SS-EPI DWI for R5 (**e**) and R6 (**f**). FOCUS MUSE shows a significant improvement in staging accuracy compared to SS-EPI DWI (*p* < 0.001). DWI, Diffusion-weighted imaging; FOCUS MUSE, Field-of-view optimized and constrained undistorted single-shot multiplexed sensitivity-encoding; R, reader; SS-EPI, Single-shot echo-planar imaging

**Fig. 5:**
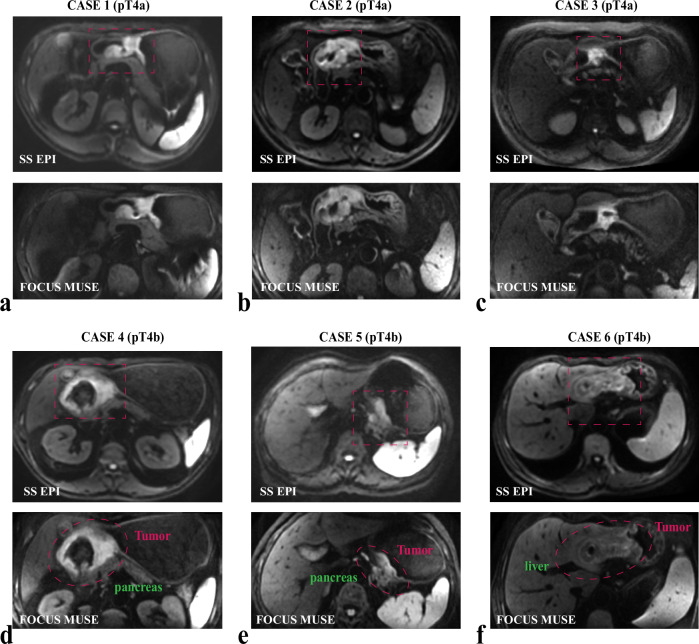
Representative cases of pathologically confirmed T4a (**a**–**c**), surgically confirmed T4b (**d**), and pathologically confirmed T4b (**e**, **f**) gastric cancer are presented. **a**–**c** All three cases demonstrate a distinct separation between the tumor and the pancreas, with no evidence of pancreatic invasion, consistent with T4a staging. **d** Case 4 depicts a tumor located in the gastric antrum. Intra-operative findings revealed tumor invasion into the pancreatic head, making the lesion unresectable. On the SS-EPI DWI, the presence of gaps between the tumor and the pancreas led to an incorrect staging of T4a. In contrast, FOCUS MUSE accurately identified the lesion as T4b. **e** Case 5 illustrates a lesion on the lesser curvature of the gastric body. Surgical findings confirmed pancreatic tail invasion, requiring a combined gastrectomy and distal pancreatectomy, with postoperative pathology verifying tumor infiltration into the pancreas. FOCUS MUSE DWI offered improved visualization of the tumor’s invasive extent, enabling a correct diagnosis compared to SS-EPI DWI. **f** In Case 6, the tumor exhibited liver invasion, as identified by both SS-EPI and FOCUS MUSE DWI. The patient underwent a combined gastrectomy and left hemihepatectomy. Tumor (red) and adjacent organs (green) are annotated for clarity. DWI, Diffusion-weighted imaging; FOCUS-MUSE, Field-of-view optimized and constrained undistorted single-shot multiplexed sensitivity-encoding; SS-EPI, Single-shot echo-planar imaging

Inter-reader agreement for both FOCUS-MUSE and SS-EPI DWI was excellent for Reader 5 and Reader 6 (κ = 0.836 and 0.898). Intra-reader consistency was also excellent, with values ranging from 0.882 to 0.929 (Table 3). Detailed confusion matrix results are summarized in Fig. 4a–d. Lesion detection was higher with FOCUS-MUSE than with SS-EPI (Reader 5, 0.930 *versus* 0.883, *p* = 0.031; Reader 6, 0.891 *versus* 0.844, *p* = 0.031). When evaluating T staging using FOCUS-MUSE and SS-EPI DWI, the diagnostic accuracy for two radiologists was as follows: 0.648 (0.562–0.726) *versus* 0.445 (0.362–0.532) and 0.633 (0.547–0.711) *versus* 0.477 (0.392–0.563), respectively, with both differences being statistically significant (*p* < 0.001) (Fig. 4e, f). Detailed macro-averaged and per-class sensitivity and specificity are reported in Supplementary Tables S3 and S4. Figures 3 and 5 provide detailed assessments for T1–T3 and T4a/b staging, respectively.

**Table 3:** Intra-reader and inter-reader agreement for T stage

T staging	Weighted kappa		
	Intra-rater (R5)	Intra-rater (R6)	Inter-rater
SS-EPI DWI	0.882	0.898	0.836
FOCUS-MUSE DWI	0.929	0.916	0.898

*DWI* Diffusion-weighted imaging, *FOCUS-MUSE* Field-of-view optimized and constrained undistorted single-shot multiplexed sensitivity-encoding, *SS-EPI* Single-shot echo-planar imaging

## Discussion

In the pursuit of precision in gastric cancer staging to support personalized treatment approaches, diffusion-weighted imaging (DWI) has emerged as a promising noninvasive modality for assessing tumor invasiveness [18]. In this context, our study evaluated the potential of FOCUS-MUSE DWI to improve image quality and diagnostic accuracy in determining tumor depth of invasion in gastric cancer. Our findings demonstrated that FOCUS-MUSE DWI significantly outperformed SS-EPI DWI in both subjective and objective assessments of image quality (all *p* < 0.001). Furthermore, the minimal bias observed in apparent diffusion coefficient (ADC) measurements underscores the reliability and robustness of FOCUS-MUSE DWI. Importantly, FOCUS-MUSE DWI demonstrated significantly higher T-staging accuracy than SS-EPI DWI (*p* < 0.001), thereby reinforcing its potential to guide clinical decision-making and treatment planning.

Despite advancements in DWI for gastric cancer, practical challenges remain [19]. The complex anatomy of the stomach, coupled with air-related, peristaltic, and respiratory motion, can lead to susceptibility artifacts, image distortion, and signal loss, particularly with conventional single-shot echo-planar imaging techniques. These limitations can compromise lesion conspicuity and boundary sharpness, thereby impeding accurate staging. To address these intrinsic challenges of gastric DWI, several protocol optimizations were applied in this study. The administration of antispasmodic agents suppressed gastrointestinal motility, thereby improving image stability during diffusion acquisition. In addition, selective use of the prone position for distal gastric tumors allowed the air-fluid interface to shift away from the lesion, thereby reducing susceptibility-related artifacts and enhancing lesion conspicuity. These preparation strategies may have contributed to improved image quality in our cohort and could limit direct generalizability to centers that do not routinely use antispasmodics and/or prone positioning. Under these optimized imaging conditions, our results suggest that FOCUS-MUSE DWI mitigates several limitations inherent to conventional DWI. By integrating the advantages of reduced field-of-view and multi-shot techniques, FOCUS-MUSE DWI significantly improved image quality while reducing artifacts. These improvements are critical in gastric cancer imaging, where artifacts frequently affect lesion detection. The significantly higher SNR and CNR observed with FOCUS-MUSE DWI in our study were consistent with findings from Wang et al [17] and Bai et al [16], who demonstrated that FOCUS-MUSE DWI effectively enhances pancreatic and orbital ophthalmopathy image quality while preserving diagnostic information. Specifically, FOCUS-MUSE DWI demonstrated moderate effect sizes for SNR and CNR across readers (Cohen *d* = 0.42–0.63), suggesting practical relevance of the observed improvements. The improved lesion conspicuity and overall image quality achieved with FOCUS-MUSE DWI are essential for accurate gastric cancer staging.

ADC has emerged as a critical parameter for assessing gastric cancer noninvasively [7]. Recent studies have reported its potential in predicting staging, neoadjuvant therapy response, and prognosis [8, 20–22]. In our study, quantitative analysis revealed that ADC values derived from FOCUS-MUSE DWI were slightly higher than those from SS-EPI DWI, with excellent intra- and inter-reader reliability. Although there was a statistically significant difference in ADC values between the two sequences, the corresponding effect sizes were small (Cohen *d* = 0.29–0.38), indicating that the magnitude of ADC variation is likely of limited clinical relevance. Additionally, the median ADC values for both sequences were approximately 1.3 × 10⁻³ mm²/s, which deviate slightly from those reported in previous studies [23, 24]. This discrepancy may be attributed to differences in acquisition techniques, *b*-values, and study populations, which should be considered when comparing ADC values across studies [18].

Accurate T-staging is a cornerstone of gastric cancer evaluation, as it directly influences clinical decision-making and therapeutic strategies [19]. From a clinical workflow perspective, FOCUS-MUSE DWI did not require additional post-processing steps and was reconstructed inline. The modest increase in acquisition time is unlikely to substantially affect examination throughput in routine practice. Our data suggest that FOCUS-MUSE DWI significantly improves the overall accuracy of T staging compared with SS-EPI DWI. The enhanced image quality provided by FOCUS-MUSE DWI allowed for better delineation of lesions and gastric wall layers, thereby enabling more precise differentiation of early-stage (T1) tumors. Furthermore, FOCUS-MUSE DWI demonstrated improvements in the accuracy of T4b staging by providing better visualization of tumor margins and detecting subtle invasions into adjacent organs, particularly the pancreas. This improvement has critical implications for surgical planning, as T4b tumors often necessitate multivisceral resection or adjuvant therapy to achieve tumor downstaging, ultimately increasing the likelihood of achieving an R0 resection [2]. Given the limited number of T4b cases, the study was not powered for definitive subgroup conclusions; therefore, findings related to advanced-stage disease should be interpreted with caution and considered hypothesis-generating. High intra- and inter-reader agreement further emphasizes the reliability and reproducibility of FOCUS-MUSE DWI in gastric cancer staging. The study was designed to compare DWI techniques and does not represent comprehensive clinical staging. Accordingly, the incremental diagnostic value of DWI over standard multiparametric MRI or contrast-enhanced CT remains to be determined in future comparative studies. Because all examinations were performed on a single-vendor 3-T system, external validation across other vendors and field strengths is warranted.

Our study had limitations. First, the sample size for advanced T4b staging was relatively small, which may have introduced selection bias and limited the generalizability of our findings. Second, this was a single-center study, and we did not perform a head-to-head comparison with CT or include MRI sequences beyond DWI; therefore, the incremental value of MRI over CT and of multi-sequence MRI over DWI alone could not be determined. Third, all MRI examinations were performed on a single 3-T system from a single vendor. Although this ensured protocol consistency, the performance of FOCUS-MUSE DWI may differ on 1.5-T systems or across different vendors, which may limit the generalizability of our findings. Fourth, MRI was obtained based on predefined referral indications rather than being performed routinely for all gastric cancer patients, introducing potential selection bias despite prospective consecutive accrual. Finally, nodal (N) staging was not assessed; future work will prospectively evaluate FOCUS-MUSE DWI for N staging with node-by-node imaging-pathology correlation and comparison with CT and other MRI sequences.

In conclusion, FOCUS-MUSE DWI demonstrated significant advantages over SS-EPI DWI in gastric cancer imaging, providing higher image quality and improved diagnostic accuracy for T-staging. These findings underscore the potential of FOCUS-MUSE DWI to enhance clinical decision-making in gastric cancer management.

## Supplementary information

**Additional File 1:** **Table** **S1**. Readers’ characteristics. **Table** **S2**. Acquisition parameters of DWI sequences. **Table** **S3**. T-staging performance by reader and sequence. **Table** **S4**. Per-class T-staging metrics by reader and sequence. **Fig.** **S1**. Bland-Altman plot represents intra-reader reproducibility for Reader 3 and Reader 4 for SNR, CNR, and ADC values. The center line (blue) represents the mean of differences, the top line (orange) shows the upper 95% limit of agreement, and the bottom line (orange) shows the lower 95% limit of agreement. *Reader’s second-time results. **Fig.** **S2**. Bland-Altman plot represents inter-reader reproducibility between readers 3 and 4 for SNR, CNR, and ADC values. The center line (blue) represents the mean of differences, the top line (orange) shows the upper 95% limit of agreement, and the bottom line (orange) shows the lower 95% limit of agreement. **Fig.** **S3**. Bland-Altman plots and paired comparisons of ADC values between SS-EPI DWI and FOCUS MUSE DWI. (**a**, **b**) The mean differences between FOCUS-MUSE and SS-EPI DWI were 0.05 for R3 (**a**) and 0.03 for R4 (**b**). The plots demonstrate a minor systematic bias indicating good concordance between the two methods. (**c**, **d**) Quantitative ADC values were higher for FOCUS MUSE DWI compared to SS-EPI DWI for both readers (*p* < 0.0001 for R3; *p* < 0.001 for R4).

## Data Availability

The datasets generated and analyzed during the current study are not publicly available due to institutional restrictions and patient confidentiality policies. However, de-identified data may be made available from the corresponding author upon reasonable request and with appropriate institutional approvals.

## Abbreviations

ADC: Apparent diffusion coefficient
CNR: Contrast-to-noise ratio
CT: Computed tomography
DWI: Diffusion-weighted imaging
FOCUS: Field-of-view optimized and constrained undistorted single-shot
MRI: Magnetic resonance imaging
MUSE: Multiplexed sensitivity-encoding
ROI: Regions of interest
SD: Standard deviation
SNR: Signal-to-noise ratio
SS-EPI: Single-shot echo-planar imaging
