# Supplementary material for: Improved image quality and T-staging accuracy using FOCUS-MUSE DWI in gastric cancer: a prospective comparison with SS-EPI
Source: Eur Radiol Exp. 2026 Jun 17;10:90. doi: 10.1186/s41747-026-00755-6 (PMC13275986; doi:10.1186/s41747-026-00755-6)
Supplement: Supplementary file 1 — Additional File 1: Table S1. Readers’ characteristics. Table S2. Acquisition parameters of DWI sequences. Table S3. T-staging performance by reader and sequence. Table S4. Per-class T-staging metrics by reader and sequence. Fig. S1. Bland-Altman plot represents intra-reader reproducibility for Reader 3 and Reader 4 for SNR, CNR, and ADC values. The center line (blue) represents the mean of differences, the top line (orange) shows the upper 95% limit of agreement, and the bottom line (orange) shows the lower 95% limit of agreement. *Reader’s second-time results. Fig. S2. Bland-Altman plot represents inter-reader reproducibility between readers 3 and 4 for SNR, CNR, and ADC values. The center line (blue) represents the mean of differences, the top line (orange) shows the upper 95% limit of agreement, and the bottom line (orange) shows the lower 95% limit of agreement. Fig. S3. Bland-Altman plots and paired comparisons of ADC values between SS-EPI DWI and FOCUS MUSE DWI. (a, b) The mean differences between FOCUS-MUSE and SS-EPI DWI were 0.05 for R3 (a) and 0.03 for R4 (b). The plots demonstrate a minor systematic bias indicating good concordance between the two methods. (c, d) Quantitative ADC values were higher for FOCUS MUSE DWI compared to SS-EPI DWI for both readers (p < 0.0001 for R3; p < 0.001 for R4). [file 41747_2026_755_MOESM1_ESM.pdf]

Improved image quality and T-staging accuracy using FOCUS-MUSE DWI in gastric cancer: a prospective comparison with SS-EPI

ELECTRONIC SUPPLEMENTARY MATERIAL

Table S1 Readers' characteristics

Reader	Roles in study	Years of experience	Seniority	MRI reads per year
Reader 1	Qualitative assessments	3	Junior	300
Reader 2	Qualitative assessments	5	Junior	500
Reader 3	Quantitative assessments	4	Junior	400
Reader 4	Quantitative assessments	5	Junior	500
Reader 5	T-staging	10	Senior	1000
Reader 6	T-staging	15	Senior	1500

Table S2 Acquisition parameters of DWI sequences

Parameter	FOCUS-MUSE DWI	SS-EPI DWI
Orientation	Axial	Axial
Breathing schemes	Respiratory trigger	Respiratory trigger
TR (ms)	4615	4615
TE (ms)	54.3	57.9
Number of shots	2	1
FOV (mm)	320 × 160	400 × 320
Matrix size	160 × 80	128 × 128
Slice thickness/gap (mm)	5/1	5/1
Number of slices	25	25
b-values (s/mm^2)	50, 800	50, 800

DWI Diffusion-weighted imaging, *FOCUS-MUSE* Field-of-view optimized and constrained undistorted single-shot multiplexed sensitivity-encoding, *SS-EPI* Single-shot echo-planar imaging, *TE* Echo time, *TR* Repetition time.

Table S3 T-staging performance by reader and sequence

Reader	Sequences	Detection rate	Macro sensitivity	Macro specificity
	SS-EPI DWI	0.883	0.399	0.877
R5	FOCUS-MUSE DWI	0.930	0.628	0.921
	SS-EPI DWI	0.844	0.410	0.896
R6	FOCUS-MUSE DWI	0.891	0.616	0.929

Detection rate, proportion of cases with a non-zero prediction (T1–T4b). Macro-averaged sensitivity/specificity, mean of one-*versus*-rest classwise metrics across T1–T4a and T4b. *DWI* Diffusion-weighted imaging, *FOCUS-MUSE* Field-of-view optimized and constrained undistorted single-shot multiplexed sensitivity-encoding *SS-EPI* Single-shot echo-planar imaging

Table S4 Per-class T-staging metrics by reader and sequence

		T1	T2	T3	T4a	T4b
Sensitivity						
	SS-EPI DWI	0.208	0.083	0.544	0.560	0.600
R5	FOCUS MUSE DWI	0.542	0.417	0.702	0.680	0.800
	SS-EPI DWI	0.125	0.250	0.596	0.680	0.400
R6	FOCUS MUSE DWI	0.417	0.417	0.684	0.760	0.800
Specificity						
	SS-EPI DWI	0.971	0.914	0.732	0.777	0.992
R5	FOCUS MUSE DWI	0.981	0.948	0.831	0.854	0.992
	SS-EPI DWI	0.981	0.948	0.775	0.786	0.992
R6	FOCUS MUSE DWI	0.981	0.957	0.873	0.845	0.992

DWI Diffusion-weighted imaging, *FOCUS MUSE* Field-of-view optimized and constrained undistorted single-shot multiplexed sensitivity-encoding, *R* Reader, *SS-EPI* Single-shot echo-planar imaging.

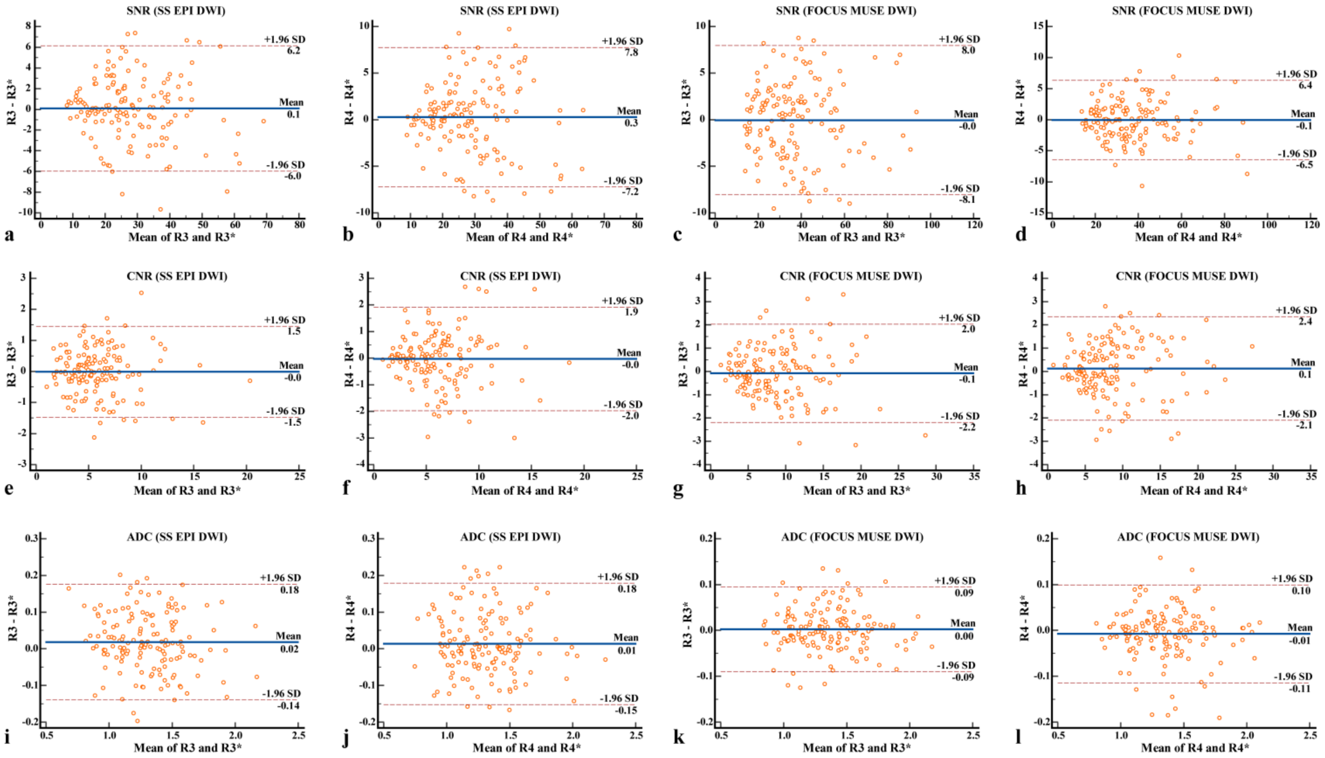

Fig. S1 Bland-Altman plot represents intra-reader reproducibility for Reader 3 and Reader 4 for SNR, CNR, and ADC values. The center line (blue) represents the mean of differences, the top line (orange) shows the upper 95% limit of agreement, and the bottom line (orange) shows the lower 95% limit of agreement. *Reader's second-time results. *ADC* Apparent diffusion coefficient, *CNR* Contrast-to-noise ratio, *DWI* Diffusion-weighted imaging, *FOCUS MUSE* Field-of-view optimized and constrained undistorted single-shot multiplexed sensitivity-encoding, *SNR* Signal-to-noise ratio, *SS-EPI* Single-shot echo-planar imaging.

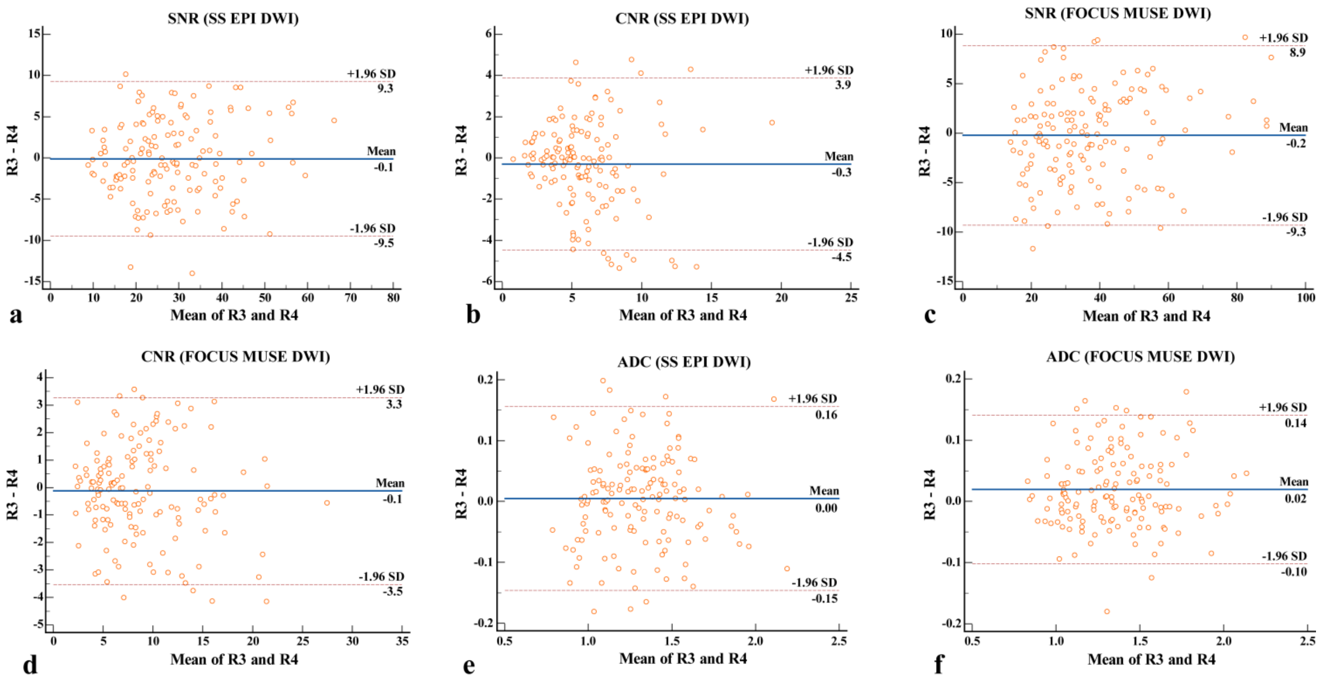

Fig. S2 Bland-Altman plot represents inter-reader reproducibility between readers 3 and 4 for SNR, CNR, and ADC values. The center line (blue) represents the mean of differences, the top line (orange) shows the upper 95% limit of agreement, and the bottom line (orange) shows the lower 95% limit of agreement. *ADC* Apparent diffusion coefficient, *CNR* Contrast-to-noise ratio, *DWI* Diffusion-weighted imaging, *FOCUS MUSE* Field-of-view optimized and constrained undistorted single-shot multiplexed sensitivity-encoding, *R* Reader, *SNR* Signal-to-noise ratio, *SS-EPI* Single-shot echo-planar imaging.

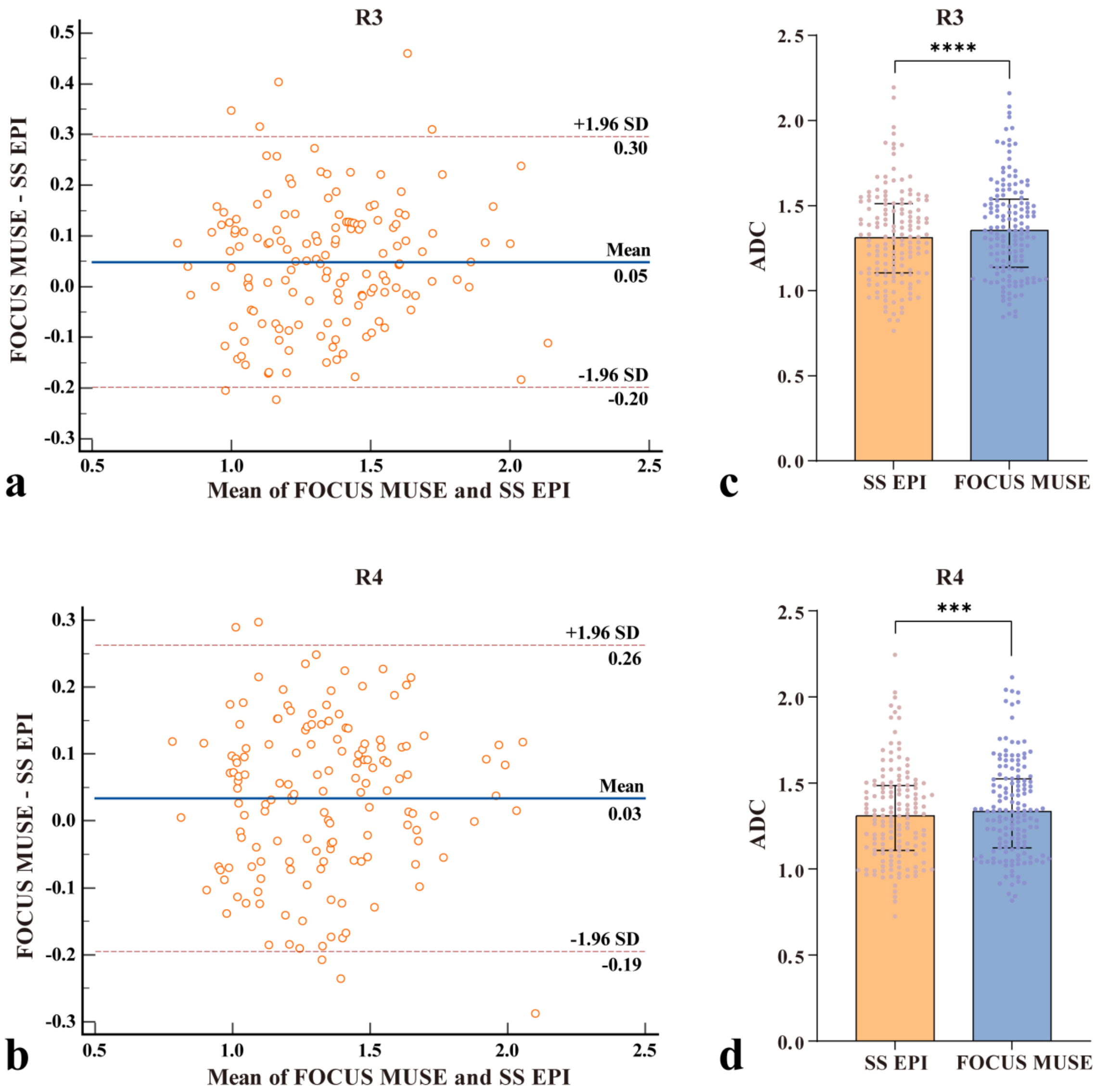

Fig. S3 Bland-Altman plots and paired comparisons of ADC values between SS-EPI DWI and FOCUS MUSE DWI. (**a**, **b**) The mean differences between FOCUS-MUSE and SS-EPI DWI were 0.05 for R3 (**a**) and 0.03 for R4 (**b**). The plots demonstrate a minor systematic bias indicating good concordance between the two methods. (**c**, **d**) Quantitative ADC values were higher for FOCUS MUSE DWI compared to SS-EPI DWI for both readers ($p < 0.0001$ for R3; $p < 0.001$ for R4). *ADC* Apparent diffusion coefficient, *DWI* Diffusion-weighted imaging, *FOCUS MUSE* Field-of-view optimized and constrained undistorted single-shot multiplexed sensitivity-encoding, *R* Reader, *SS-EPI* Single-shot echo-planar imaging.
